# Early Postnatal Experience Modifies Activation of the Pituitary Testicular Complex in Male House Mice (*Mus Musculus*) Exposed to the Odor of Receptive Con- and Heterospecific Females

**DOI:** 10.1134/S0012496623700539

**Published:** 2023-10-13

**Authors:** E. V. Kotenkova, E. V. Kuznezova, A. N. Maltsev, A. V. Ambaryan

**Affiliations:** Severtsov Institute of Problems of Ecology and Evolution, Russian Academy of Sciences, 119071 Moscow, Russia

**Keywords:** early experience, maternal environment, testosterone level, olfactory signals, isolating mechanisms

## Abstract

For the first time, it was shown that activation of the pituitary–testicular complex in male house mice exposed to the odor of receptive females of their own and closely related species was modified under the influence of early postnatal experience and the maternal environment. We have confirmed associated formation of behavioral and physiological mechanisms of precopulatory isolation in early ontogenesis. The serum levels of free testosterone in males of closely related species *M. spicilegus* and *M. m. wagneri* differ, it is significantly lower in mound-building mice. In males fostered by a conspecific female, the level of free testosterone was significantly lower when exposed to a heterospecific female odor in comparison with a conspecific odor. The rearing of *M. m. wagneri* males by females of a closely related species led to a decrease in the testosterone response caused by exposure to female chemosignals (both con- and heterospecific) and to the absence of differences in the serum level of free testosterone when exposed to the odor of a female of their own or closely related species. These results indicate that the rearing conditions had a significant influence on the formation of hormonal mechanisms of reproductive isolation.

In mammals, including house mice, the response to the species-specific odor is formed in early postnatal ontogenesis as a result of imprinting or other forms of learning, which subsequently affects various behavioral manifestations determining the choice of a conspecific sexual partner, i.e., the formation of precopulatory isolation mechanisms [1]. One of the mechanisms of reproductive isolation that prevents mating with heterospecific individuals in mammals, including house mice, is the response to olfactory signals, which may be due to differences in the composition of signaling compounds (pheromones) present in the urine of closely related species [2, 3] and causing certain physiological reactions in conspecifics [4]. Exposure to conspecific females triggers a series of behavioral and physiological responses that stimulate sexual behavior and usually lead to mating [4]. The pituitary–testicular complex in males is activated by direct contact with receptive conspecific females or by presentation with the odor of such females [5–8]. In males of two sympatric species, *Mus musculus* (L. 1758) and *M. spicilegus* (Petenyi 1882), exposure to a receptive female of the same species, as opposed to a female of a closely related species, caused a rise in plasma testosterone levels [9]. One of the main approaches to studying the influence of early experience on subsequent behavior and response to con- and heterospecific stimuli is the rearing of offspring by members of another species [1, 10]. This may lead to a preference for the odor of individuals of the foster species over the odor of their own species. The rearing of the young by foster parents of a closely related species modified the assortativity of choice of con- and heterospecific odors in house and mound-building mice. In particular, *M. spicilegus* reared by *M. musculus musculus* females had a preference for the odor of the foster species females; *M. m. wagneri* reared by *M. spicilegus* and *M. spicilegus* reared by *M. m. wagneri* did not have a preference for the odor of females of either their own or the foster species [11]. According to the hypothesis proposed by Cheal [12], the behavioral and physiological responses to odors are autonomous, and only behavioral responses can be modified under the influence of early olfactory experience, while physiological responses do not change. We are not aware of any works concerning the properties of the pituitary–testicular complex activation in male house mice and other rodents reared by members of a closely related species in response to exposure to the odor of receptive conspecific females and females of the rearing species, which could confirm or refute the stated hypothesis.

The goal of this work was to reveal the effects of early experience and the maternal environment on the formation of physiological mechanisms of precopulatory isolation in house mice and to find out whether they developed autonomously or in association with behavioral mechanisms of reproductive isolation. For this purpose, we compared the hormonal response (free serum testosterone (FST) levels) to the odor of receptive conspecific females and females of the foster species in *M. m. wagneri* males reared in the families of the closely related species *M. spicilegus*. For the first time, we have demonstrated that early postnatal experience and maternal environment modify the activation of the pituitary–testicular complex in males exposed to the odor of con- and heterospecific females. We have confirmed that the formation of behavioral and physiological mechanisms of precopulatory isolation during the early ontogenesis occurred in association.

Experiments were performed in male house mice reared by members of a closely related species. At the age of 2–3 days, males of *M. m.* *wagneri* were transferred into families of the closely related species *M. spicilegus*. Female offspring were left in their families. At the age of 40 days, young males were separated from adults into same-sex groups, each litter in their own cage. At the age of 60 days, the mice were placed individually in glass chambers with metal lids, where the subsequent experiments were conducted. Altogether, the experiments involved 29 *M. m.* *wagneri* males reared by conspecific females, 13 *M. m.* *wagneri* males reared by *M. spicilegus* females, and 23 *M. spicilegus* males reared by conspecific females.

All exposures were carried out at the same time of the day (from 11 a.m. to 1 p.m.). Each male was exposed to urine twice (once from a conspecific female, and once from the foster species) with an interval of 8 or 16 days. The animals were exposed to 20 μL urine for 30 min in their home cage, and a blood sample of no more than 0.15 mL was collected from the retro-orbital sinus [13]. Control animals were exposed to physiological saline (Table 1). Blood was collected in clean Eppendorf tubes and centrifuged at 3000 *g* for 10 min to separate the serum, which was transferred into fresh tubes and stored at –20°C until the analysis (no longer than 1 month after blood collection). The concentration of free testosterone (pg/mL) was measured by solid-phase ELISA using a reagent kit purchased from DRG Diagnostics (Germany; cat. no. EIA2924) according to the manufacturer’s instructions. The optical density of solution in the plate wells was measured at 450 nm using an iMark microplate absorbance reader (Bio-Rad, United States).

**Table 1.** Number of odor presentations and FST levels in the experimental groups

Number of odor presentations; FST levels (pg/mL)	Experimental groups*							
	*Spicilegus*/ *spicilegus—* physiological saline	*Spicilegus*/ *spicilegus*— urine of *M. wagneri*	*Spicilegus*/ *spicilegus*— urine of *M. spicilegus*	*Wagneri*/*wagneri—* physiological saline	*Wagneri*/*wagneri—* urine of *M. wagneri*	*Wagneri*/*wagneri —* urine of *M. spicilegus*	*Wagneri*/ *spicilegus*— urine of *M. wagneri*	*Wagneri*/ *spicilegus—* urine of *M. spicilegus*
Number of presentations	8	6	6	8	13	9	8	7
Minimum/ maxum/median FST levels	0.020/0.160/ 0.095	0.120/23.590/ 2.150	0.130/100.000/2.740	0.040/100.000/ 61.795	0.340/100.000/ 85.200	0.230/69.940/ 11.320	0.090/92.070/ 6.280	0.110/95.070/ 0.760

*Wagneri/wagneri stands for *M. m. wagneri* reared by conspecific females; *wagneri/spicilegus*, *M. m. wagneri* reared by *M. spicilegus*; *spicilegus/spicilegus,* *M. spicilegus* reared by conspecific females*.* The group names list with a dash the recipient male type and the odor signal type.

Statistical analysis of the data was carried out using Xlstat 2019.2.2 software. Intergroup comparisons of FST proportions from the maximum possible (detected) concentration of 100 units were performed using the formula *N* × 100, where 100 was the maximum detected concentration of free testosterone, and *N* was the sample size. Proportions were compared using the χ^2^ test. Pairwise comparisons of proportions were performed using the Marascuillo procedure. The significance threshold for pairwise comparisons was α = 0.05.

The results of pairwise comparison of FST proportions from the maximum possible level for each group showed that *M. spicilegus* males reared by conspecific females had the lowest FST in response to physiological saline among all groups (χ^2^ statistics, 1271.612, *P* < 0.0001; Table 2); that is, they had a significantly lower background FST level than *M. m. wagneri* males. FST in *M. spicilegus* males increased significantly when they were exposed to urine of both conspecific females and *M. m. wagneri* females. The relatively low FST levels in *M. spicilegus* males in the absence of social context due to the presence of female individuals can probably be explained by higher corticosterone levels in these exoanthropic species. This may be related, on the one hand, to selection and evolutionary adaptations determined by the properties of their habitat: open steppe biotopes and agrocenoses, and on the other hand, to the features of their group social structure and the mating system that can be considered facultatively monogamous, in contrast to the social structure and predominantly polygamous mating system in *M. musculus* [14]. Mound-building mice are highly territorial, and contacts between stranger individuals (both males and females) may result in extreme aggression. This may have contributed to the evolutionary fixation of the relatively high corticosterone levels in males of this species. At the same time, FST levels in *M. m. wagneri* males reared by conspecific females were high when exposed to physiological saline and comparable to the levels caused by exposure to the odor of a conspecific female. Heterospecificity of the foster female or the odor signal, as well as their combination, caused a lower FST response in different groups of *M. m. wagneri* males. The differences in FST levels between these groups of males were not significant. Importantly, exposure to a heterospecific odor signal caused a significantly lower FST response in males reared by a conspecific females than a conspecific odor signal. However, this response did not differ significantly from FST levels in *M. m. wagneri* males reared by a *M. spicilegus* female, both when exposed to a heterospecific odor and to a conspecific odor (although a decrease in FST was less pronounced in the latter case). Heterospecificity of both the rearing female and the odor signal type had the strongest diminishing effect on FST levels in *M. m. wagneri* males reared by *M. spicilegus* females, making them close to FST levels in *M. spicilegus* males exposed to a conspecific signal. That is, the rearing of *M. m. wagneri* males by females of a different closely related species, on the one hand, led to a decrease in the males’ testosterone response to chemical signals of females (both hetero- and conspecific) and on the other hand, leveled out the differences in the FST response to the odor of a con- or heterospecific female. We suppose that this was related to modified perception of species identity of individuals of the opposite sex in *M. m. wagneri* males reared by *M. spicilegus* females. Since the behavioral patterns exhibited by their foster mother were not fully consistent with species-specific ones, *M. m. wagneri* males reared by a closely related species probably developed a less definite and/or more generalized olfactory image of species specificity. As a result, their response to chemosignals from individuals of the opposite sex could become less pronounced (relatively low FST) and less species-specific (little difference in responses to con- and heterospecific female odors).

**Table 2.** Pairwise comparison of the groups of *M. spicilegus* and *M. wagneri* males by FST proportions

Male groups analyzed (recipient male species/rearing female species—odor signal—FST proportion	Male groups determined by combination of species specificity of the rearing female and the odor signal type		Proportion	Absolute difference of proportions (test statistics)	Critical value of test statistics (*r*)	Significance of differences
	Male species/rearing female species	Odor (urine of receptive female)				
*Spicilegus/spicilegus—*physiological saline—0.0013	*Spicilegus/spicilegus*	*Wagneri*	0.0600	0.059	0.037	**Yes**
	*Spicilegus/spicilegus*	*Spicilegus*	0.1883	0.187	0.060	**Yes**
*Spicilegus/spicilegus*—urine of *spicilegus*-0.1883	*Wagneri/wagneri*	*Wagneri*	0.5685	0.380	0.079	**Yes**
	*Wagneri/wagneri*	*Spicilegus*	0.2222	0.034	0.079	No
	*Wagneri/spicilegus*	*Wagneri*	0.2763	0.088	0.084	**Yes**
	*Wagneri/spicilegus*	*Spicilegus*	0.2100	0.022	0.083	No
*Wagneri/wagneri*—physiological saline—0.5338	*Wagneri/wagneri*	*Wagneri*	0.5685	0.035	0,084	No
*Wagneri/wagneri*—urine of *wagneri—*0.5685	*Wagneri/wagneri*	*Spicilegus*	0.2222	0.346	0.073	**Yes**
	*Wagneri/spicilegus*	*Wagneri*	0.2763	0.292	0.079	**Yes**
	*Wagneri/spicilegus*	*Spicilegus*	0.2100	0.358	0.077	**Yes**
*Wagneri/wagneri—*urine of *spicilegus*—0.2222	*Wagneri/spicilegus*	*Wagneri*	0.2763	0.054	0.079	No
	*Wagneri/spicilegus*	*Spicilegus*	0.2100	0.012	0.078	No
*Wagneri/spicilegus*—urine of *wagneri*—0.2763	*Wagneri/spicilegus*	*Spicilegus*	0.2100	0.066	0.083	No

To sum up, we should note that the background FST levels were different in males of two closely related species, *M. spicilegus* (exoanthropic species) and *M. m. wagneri* (prone to synanthropy but also inhabiting open biotopes): they were significantly lower in mound-building mice. Early olfactory experience and changes in the maternal environment modify the species-specific activation of the pituitary–testicular complex in *M. m. wagneri* males exposed to the urine odor of conspecific females and of foster species. This is consistent with our previous results, which demonstrated an opposite character of neuron activation in the accessory olfactory bulb (AOB) of *M. m. wagneri* males reared by *M. spicilegus* females and of males reared by conspecific females. The maternal environment, including odor, had a more pronounced effect on the MRI signal level in the AOB of mature males than the genetic relationship between the recipient and the donor of the odor [11]. The data from this study suggest that the rearing conditions have a significant effect on the development of hormonal mechanisms of reproductive isolation in house mice, and together with our previous results, they indicate that behavioral, neuronal, and hormonal responses in males are modified by the early experience and maternal environment. These modifications occurred in the same direction, which speaks against the hypothesis about autonomous formation of behavioral and physiological mechanisms underlying reproductive isolation in house mice of the species studied.
